# Clinical Information Extraction From Notes of Veterans With Lymphoid Malignancies: Natural Language Processing Study

**DOI:** 10.2196/63908

**Published:** 2025-10-16

**Authors:** Lu He, Matthew R Moldenhauer, Kai Zheng, Helen Ma

**Affiliations:** 1 Zilber College of Public Health, University of Wisconsin-Milwaukee, Milwaukee, WI, United States; 2 School of Medicine, University of California, San Diego, San Diego, CA, United States; 3 Department of Informatics, Donald Bren School of Information and Computer Science, University of California, Irvine, Irvine, CA, United States; 4 Department of Emergency Medicine, University of California, Irvine, Irvine, CA, United States; 5 Veterans Affairs Health System, 5901 E 7th Street, Long Beach, CA, 90822, United States, 1 5628268000; 6 School of Medicine, University of California Irvine, Irvine, CA, United States

**Keywords:** natural language processing, NLP, clinical informatics, rare cancer, developing, validating, clinical information extraction, veterans, lymphoid malignancies, clinical documentation, non-Hispanic White, non-Hispanic Black

## Abstract

**Background:**

Clinical natural language processing (cNLP) techniques are commonly developed and used to extract information from clinical notes to facilitate clinical decision-making and research. However, they are less established for rare diseases such as lymphoid malignancies due to the lack of annotated data as well as the heterogeneity and complexity of how clinical information is documented. In addition, there is increasing evidence that cNLP techniques may be prone to biases embedded in clinical documentation or model development. These biases can result in disparities in performance when extracting clinical information or predicting patient outcomes.

**Objective:**

This study aims to report the development and validation of a cNLP pipeline that extracts clinical information such as performance status, staging, and diagnosis, as well as less common information such as substance use and military environmental exposures, from the clinical notes of veterans with lymphoid malignancies.

**Methods:**

We developed a rule-based cNLP pipeline that integrates domain expertise. We tested and compared the performance of the cNLP pipeline on notes from 2 veteran patient cohorts: one from non-Hispanic White veterans and the other from non-Hispanic Black veterans.

**Results:**

Overall, our pipeline achieved promising performance on our study data, especially for extracting entities that have standard clinical documentation, such as performance status. We also found that while the pipeline has robust performance across the two patient groups, the false-positive and false-negative rates were significantly associated with race for detecting the primary diagnosis (*P*=.001 for both); the false-negative rate was significantly associated with race for identifying substance use (*P*=.02).

**Conclusions:**

The system exhibits satisfying and comparable performance for most clinical entities of interest except for (1) the primary diagnosis and (2) substance use. Future work will address the challenges encountered in developing and deploying the cNLP pipeline on the Department of Veterans Affairs data for rare cancers and enhance the performance of cNLP systems to avoid biases.

## Introduction

Clinical notes contain rich information about patients’ conditions and disease trajectories. As it is often tedious and time-consuming for clinicians to perform a manual chart review to retrieve such information, clinical natural language processing (cNLP) has served as a crucial tool to automate the process of extracting clinical elements from unstructured notes [1]. While cNLP is an established field with mature software such as the clinical Text Analysis and Knowledge Extraction System and Clinical Language Annotation, Modeling, and Processing, there is a lack of cNLP resources for extracting information from clinical notes of patients with rare cancers [2], which may be due to the difficulty of obtaining high-quality annotation data from rare disease experts.

Lymphoid malignancies (LMs) are rare cancers with only around 145,000 patients diagnosed in the United States per year [3-7]. Among US veterans who were deployed overseas, exposure to environmental toxicants and chemical agents is a risk factor associated with LMs [8]. There is a need to capture past environmental exposures of veterans to better care for them. For example, the Promise to Address Comprehensive Toxics (PACT) Act, which passed in 2022, expands health care benefits and assists clinical research for veterans who have been exposed to environmental toxicants [9]. While the need for identifying and caring for veterans with environmental exposures increases, such information is often not well captured in structured electronic health records (EHRs). In fact, most of the clinical information that is vital to clinicians’ decision-making is embedded in free-text, unstructured clinical notes [10]. Therefore, there is a need to develop cNLP tools that are specifically tailored to and validated on clinical notes for rare cancers so that they can capture unique clinical information. To the best of our knowledge, there is no existing cNLP pipeline or model that has been developed for and validated on clinical notes of LMs.

In this study, we developed and validated a rule-based cNLP pipeline to extract diagnoses, staging, performance status, substance use, and environmental exposures from clinical notes of veterans with LMs. We further assessed whether the cNLP pipeline may be prone to biases by comparing its performance on non-Hispanic White and Black patients. Finally, we discuss the opportunities and challenges of developing cNLP pipelines for extracting information from clinical notes of patients with LMs and implications for clinical documentation.

## Methods

### Data Source and Creating the Development Set

We extracted records of veterans diagnosed with LMs—as defined by the *International Classification of Diseases for Oncology, Third Edition*—from the Department of Veterans Affairs (VA) Corporate Data Warehouse (CDW) [11] (summarized in Multimedia Appendix 1). The VA CDW includes patient records from 173 medical centers within the VA system across the United States as well as clinics in Puerto Rico, Guam, and the Philippines, capturing different practice and documentation patterns [12]. In total, 80,245 patients were identified using the *International Classification of Diseases for Oncology, Third edition*. The development set included a random sample of 287 deidentified hematology and oncology clinic notes from veterans with LMs in the VA system after removing 13 duplicates from the initial 300 notes. The hematology and oncology notes contained the most relevant clinical information and were intended to reduce annotators’ burdens.

### Clinical Entities of Interest

We annotated and extracted the following clinical entities that were known to be prognostic in the care of patients with LMs but were often inconsistently documented in a structured format using a standardized dictionary:

1. Diagnosis: primary diagnosis related to LMs
2. Substance use: alcohol, drug, and tobacco use
3. Environmental exposure: Agent Orange, Vietnam, shipyards, and Marine Corps Base Camp Lejeune according to the PACT Act
4. Staging: stage, chronic lymphocytic leukemia Rai staging and Binet staging, the multiple myeloma International Staging System, and lymphoma Ann Arbor staging
5. Performance status: performance status, Karnofsky performance status (KPS), and Eastern Cooperative Oncology Group (ECOG) performance status

### Annotation and Scoring

Clinicians annotated according to an annotation guideline, provided in Multimedia Appendix 2. The annotation guideline was continuously improved during the annotation process and aimed to capture common and recurring documentation patterns rather than a comprehensive list of documentation. Two independent medical experts annotated a random sample of 100 notes from the development set to calibrate. The interrater agreement ratio was calculated, and disagreements were discussed and resolved to finalize the annotation protocol. The annotation task was formulated as selecting a text span that indicates a clinical entity of interest and selecting the entity label (eg, stage and performance status). Therefore, each annotated entity can be represented as having the following: (1) a label *L* and (2) a text span with starting and ending indices (*i, j*). Two annotated entities *M* and *N* are considered a match if Label (M) = Label (N) ^ relaxed_match (span (M), span (N)).

We calculated the span-based *F*_1_-score for measuring the level of agreement between the two annotators, following previous studies [13-15]. The span-based *F*_1_-score is preferable in this case because it is not affected by the number of true negatives, which are prevalent in span-based annotations [16]. We calculated the metrics for span-relaxed matches, where 2 text spans need to overlap for at least two-thirds of the length of the shortest span. The microaveraged *F*_1_-score is 0.85; the detailed statistics across different entity types are presented in Table 1.

**Table 1.** Interrater agreement of 100 annotated notes in the development set.

	Precision	Recall	*F*_1_-score
Primary diagnosis	0.89	0.79	0.84
Performance status	0.90	0.87	0.88
Staging	0.90	0.98	0.94
Substance use	0.66	0.90	0.76
Environmental exposure	0.64	0.70	0.67

The clinician annotators met regularly to discuss and resolve disagreements and adjust the annotation guidelines accordingly. After reaching consensus, they proceeded to annotate the remainder of the development set, test set 1, and test set 2. The annotation task was formulated as named entity extraction, where annotators selected a text span and labeled it with a clinical entity such as diagnosis. For each note, there were multiple text spans with start and end indices and their associated clinical entity labels.

### Confirmatory Test Sets

To develop and test the cNLP pipeline, we randomly selected 1000 patients from the patient cohort.

We included all the hematology and oncology notes of these patients, and these comprised notes from nurses, social workers, and pharmacists who document a wide range of information. This aimed to ensure that the cNLP pipeline we developed would not be tuned to work only on specific note types or from specific provider types. When constructing the test sets, we used 2 steps to compile the notes. First, from the patient cohort, we randomly selected 100 notes with at least 300 characters to ensure that they are long enough to contain useful information. After annotating a few notes, we noticed that for some clinical entities, especially performance status and environmental exposures, few notes mentioned them. Therefore, besides the 100 randomly selected notes, we also included notes that contained predefined keywords (eg, “ECOG” and “Agent Orange”) so that medical experts did not need to read notes without any clinical entities of interest. In this process, the random selection of notes helped to ensure the representativeness of the notes, and the use of keyword filtering helped to increase the recall and lower the annotator burden. This method of using keywords to prefilter notes had been used in previous work to extract infrequently documented information, such as housing eviction status, from clinical notes [17].

**Figure 1.**
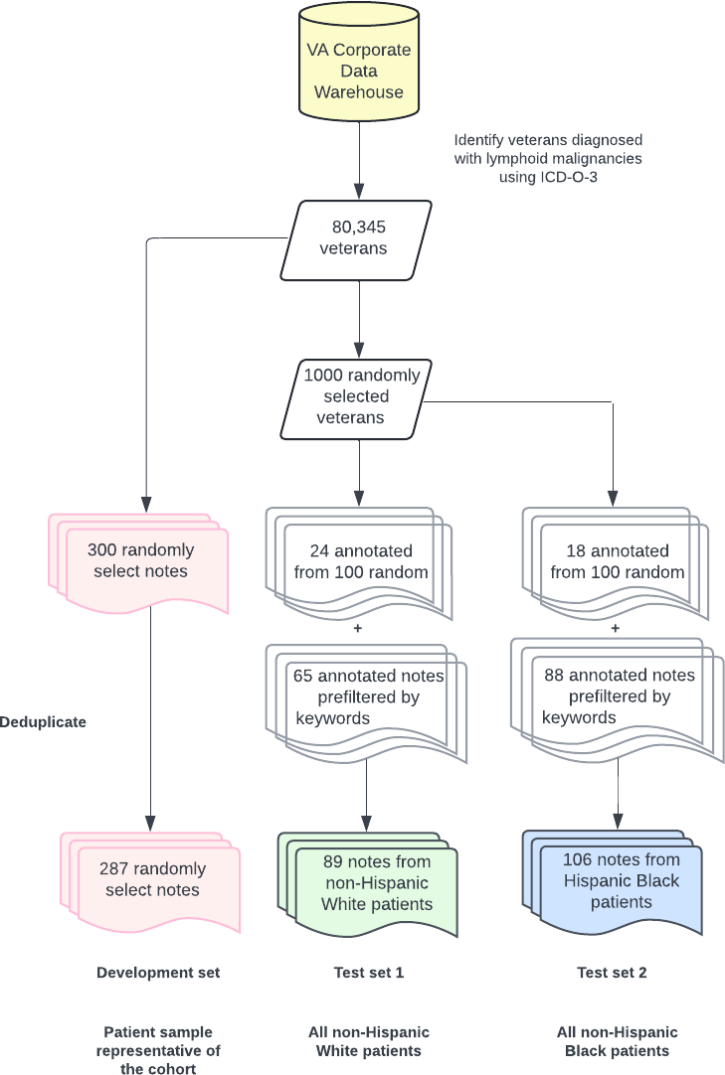
Procedure for constructing datasets for clinical natural language processing development and validation. *ICD-O-3*: *International Classification of Diseases for Oncology, Third Edition*; VA: Veterans Affairs.

The first test set (test set 1) included 200 notes from all races and ethnicities, resulting in a sample of 89 notes with clinically relevant data from randomly selected non-Hispanic White patients. Given potential differences in documentation, we added a second group enriched for non-Hispanic Black patients. The second test set (test set 2) included a sample of 200 notes from randomly selected non-Hispanic Black patients, resulting in 106 notes containing clinically relevant data. The data selection and processing procedures are shown in Figure 1.

### Development of the cNLP Pipeline

The goal of the study was to develop a cNLP pipeline that can accurately extract clinical entities of interest without extensive annotations. Therefore, we did not perform a comprehensive evaluation of pretrained language models, as most were not developed on notes from patients with LMs and fine-tuning them will require more annotated data.

We tested 2 zero-shot large language models (LLMs), FLAN-T5 by Google and Llama2 by Meta, with carefully curated prompts to extract the clinical entities of interest. We did not test OpenAI’s GPT models due to the sensitive nature of our data and the requirements of sending the input data to the commercial servers of OpenAI. The zero-shot LLMs also did not produce a satisfactory and consistent identification for the named entity recognition task, which was also observed in a recent evaluation conducted by Lu et al [18]. We found that Llama2 had difficulty following the instructions, and FLAN-T5 was only able to correctly identify 16 out of 137 incidences of performance status and 263 out of 1347 diagnosis mentions. We reviewed samples of errors and found that FLAN-T5 tended to hallucinate and made up text snippets that were not in the original clinical notes. This could be because FLAN-T5 and similar general domain LLMs were not pretrained on clinical notes or data that contain language related to performance status or LMs. Fine-tuning these LLMs will require larger datasets of clinical notes and more extensive instruction tuning and supervised fine-tuning and is beyond the scope of this study. Therefore, we decided to develop rule-based modules based on expert input. Despite the limitations of rule-based methods, they are still widely used and implemented on tasks such as identifying cognitive assessment tests and biomarkers from notes of patients with Alzheimer disease and Alzheimer disease–related dementias [19] and extracting risk factors for pancreatic cancer [20].

### Development of a Rule-Based cNLP Pipeline

The cNLP pipeline was iteratively developed by examining annotated texts in the development set as well as consulting the clinical experts on the study team. For example, “ECOG,” “KPS,” and “Performance Status” were included in the dictionary of the pipeline to capture potential mentions of performance status. Similarly, “Stage,” “Stg,” and “Rai” were included to identify mentions of staging information. “Tobacco,” “smoke,” “alcohol,” “drug,” “etoh,” and other commonly used terms were included to extract substance use mentions. For environmental exposures, we used both chemical agents, such as “Agent Orange,” and locations that are known to have toxicants, such as “Vietnam” or “shipyard.” All clinical notes were preprocessed by converting words to lowercase. Punctuation was preserved. We accounted for misspellings or format issues (eg, extra or missing spaces) by curating the regular expressions. Examples of the final regular expressions are provided in Multimedia Appendix 3.

We added special modules in the cNLP pipeline after matching regular expressions to account for the errors we observed in the training set. For the primary diagnosis and performance status, we observed that most false positives (FPs) were due to the prevalent use of templates (eg, in disability benefit questionnaires). We excluded matched diagnoses and performance status that were present in a template format (“[ ] Multiple Myeloma”; “() ECOG 1 () ECOG 2”). Excluding matches from templates reduced the number of FPs in our study sample. In addition, for the primary diagnosis, as the diagnosis process for LMs is commonly complex and often undergoes numerous laboratory tests and positron emission tomography scans, we excluded mentions of LMs that were documented as differentials or assessment plans and needed further confirmation. This step ensured that the final extracted primary diagnosis is confirmed and can be directly used for secondary analysis. For performance status, we required all possible matches of performance status to be followed by numeric values so that ambiguous expressions such as performance status for postscripts are excluded. For ECOG performance status, the numeric values must be between 0 (indicating fully active) and 5 (indicating death), and for KPS, the numeric values must be between 0 (indicating death) and 100 (indicating normal). This ensures that only text chunks that indicate performance status will be included so that FPs can be reduced.

For staging, we observed that most FPs were due to their irrelevance to our diagnosis of interests, and it was common for stages of multiple comorbidities to be documented (eg, stage II for chronic kidney disease). Therefore, we checked whether the staging information identified is within the window of a surrounding primary diagnosis. We experimented with multiple values of the size of the window to look for the primary diagnosis. The performances for extracting staging using different window sizes are provided in Multimedia Appendix 3. Overall, we observed that as the window size increased, both test sets had higher precision but lower recall. We selected a window size of 30, as the *F*_1_-scores for both test sets were optimized. The window sizes also enable more flexible configuration of the pipeline, depending on whether the priority is to extract all potentially relevant staging information (a larger window size) or to only extract highly relevant staging information (a smaller window size).

For identifying environmental exposures, we used multiple rules informed by the development data, as well as VA’s official PACT Act recommendation [9] to capture potential exposure, as exact documentation containing the names of chemical toxicants, such as Agent Orange, is limited and does not capture all potential exposures. We also included services and deployments that are potentially associated with exposures, including service in Vietnam or Camp Lejeune. Finally, we included locations that may expose patients to toxicants, including shipyards, infantry, and chemical plant factories.

### Performance Evaluation

The extracted tokens were evaluated based on the lenient matching criteria used in previous work [21,22], that is, 2 tokens were deemed as a match if they overlapped, to account for potential annotation issues and format irregularities. Precision was calculated as true positives/(true positives + FPs) and recall was calculated as true positives/(true positives + false negatives [FNs]). *F*_1_-score is (2 × precision × recall)/(precision + recall). Misclassified entities in the test set were extracted and analyzed manually by the medical experts to identify potential sources of errors to improve the performance of the cNLP pipeline.

We further assessed whether the pipeline was prone to bias on the test sets. In this study, we focused on racial bias, and in particular, potential biases between non-Hispanic White and Black patient populations. We conducted chi-square tests on FPs and FNs using the cNLP pipeline. The null hypothesis was that the cNLP performance, measured by FP and FN, was independent of the patients’ race.

### Ethical Considerations

This study was approved by the institutional review board (VA IRB #1618998 and UCI IRB #1041) at both the VA Long Beach Healthcare System and the University of California, Irvine. Research was conducted in accordance with the Declaration of Helsinki. Waivers of informed consent and HIPAA (Health Insurance Portability and Accountability Act) compliance were approved by the institutional review board.

## Results

### NLP Performance on the Test Sets

For each clinical entity, the precision, recall, and *F*_1_-score were calculated and presented in Table 2. For entities that have clear standards for documentation such as performance status, the performance is higher (*F*_1_-score>0.90). For entities that generally lack a documentation standard such as substance use or environmental exposures, we observed lower performance. In addition, the high precision and recall for some entities were possibly due to overfits of the manually tuned model, which is a common issue for rule-based pipelines.

**Table 2.** Clinical natural language processing performance on test set 1 (89 notes) and test set 2 (106 notes).

	Test set 1				Test set 2			
	Frequency	Precision	Recall	*F*_1_-score	Frequency	Precision	Recall	*F*_1_-score
Performance status	54	0.96	0.96	0.96	73	0.85	0.96	0.90
Staging	71	0.94	0.69	0.80	116	0.85	0.60	0.71
Primary diagnosis	277	0.70	0.93	0.80	485	0.60	0.85	0.70
Substance use	104	0.65	0.61	0.63	148	0.66	0.76	0.70
Environmental exposures	29	0.72	0.62	0.67	5	0.5	0.6	0.54

### Bias Assessment

The results from chi-square tests showed that the performance of the cNLP pipeline was not statistically different across test sets 1 and 2 for performance status and staging. However, the cNLP pipeline’s FN rate for extracting substance use is significantly associated with patients’ race (*χ*^2^_1_=5.9; *P*=.02). The pipeline’s FP rate for extracting the primary diagnosis is significantly associated with race (*χ*^2^_1_=10.2; *P*=.001), and the FN rate is also associated with race (*χ*^2^_1_=10.4; *P*=.001). We were unable to run the test on environmental exposures due to the small number of observations available (5/106, 4.7%). Detailed statistics and confusion matrices are provided in Multimedia Appendix 2.

### Error Analysis

Table 3 presents example snippets of FPs and FNs.

**Table 3.** Example snippets of false positive (FP) and false negative (FN)^a^.

	FP example	FN example
Performance status	“*ECOG 0‐1*”	“*PS>2*”
Staging	“Chronic kidney disease *stage 3* Multiple myeloma”	“At least *Stage IIIA*”
Primary diagnosis	“likely prostate cancer and *CLL*”	“with kappa light chain *MM*”
Substance use	“8. *Tobacco/Marijuana Use*–smoking cigarettes again”	“*crack use*”
Environmental exposures	“combat exposure”	“worked as an exterminator”

a Italicized texts indicate misalignment between natural language processing extraction and annotation.

Our qualitative analysis reveals the following two major types of errors:

1. Lack of documentation standards: many of the FNs were caused by inconsistent documentation, especially for environmental exposures. For example, while the cNLP pipeline captured common mentions such as “Agent Orange,” “service in Vietnam,” and “chemical plant factory,” exposure-related information was often documented in various ways that made it difficult for the cNLP pipeline to capture exhaustively. Furthermore, some clinicians used locations such as “shipyards” to imply potential exposures to toxicants, while some explicitly listed the chemical toxicants, such as “asbestos.”
2. Implicit association with the primary diagnosis: while our efforts in associating staging information with surrounding mentions of the primary diagnosis effectively reduced the number of FPs (ie, incorrectly identifying staging information), the pipeline also resulted in FNs where relevant staging information was not captured because there was no primary diagnosis mentioned around it. In reviewing the errors, we observed that not all LM-related staging information was explicitly documented together with the primary diagnosis.

## Discussion

### Principal Findings

We developed a rule-based cNLP pipeline to identify mentions of clinical information, including diagnoses, performance status, staging, substance use, and environmental exposures from clinical notes of veterans with LMs. The pipeline can be used in our future studies to produce weak labels, which can serve as fine-tuning datasets to improve the performance of LLMs in the domain of LMs.

In the literature, a study that reported better performance in extracting staging and substance use information from notes of patients with cancer [23]. However, the majority of these studies only used radiology and pathology notes, which were generally more structured and did not contain other clinical information unrelated to cancer [2]. In our study, the wider range of notes authored by a wide range of health providers introduced additional challenges to accurately identify clinical entities of interest due to the heterogeneity of information and documentation styles of these notes. In addition, none of the existing studies focused on rare cancers such as LMs but were related to more prevalent cancer types such as breast cancer and lung cancer. Some clinical entities and their documentation were therefore inherently different from those for LMs. For example, breast cancer documentation uses TNM staging system (where T, N, and M stand for tumor, node, and metastasis, respectively) to record staging, which is standard for most solid tumors, while LM uses histology-specific staging, such as Ann Arbor and Rai staging. The semantic rules for capturing LM-specific staging information can be used for other studies that aim to extract staging information from notes of patients with LMs. In addition, the performance of our rule-based system in extracting performance status is also comparable with the performances reported in prior studies [23,24].

### Challenges of Developing cNLP Systems for LMs

Through this study, we observed several challenges to the development of high-performing cNLP systems for LMs and other rare cancers. Future efforts in developing cNLP for rare cancers should consider addressing these challenges to improve performance, alleviate medical experts’ burden in annotation, and facilitate the use of cNLP in real-world clinical settings.

First, relevant clinical information on rare cancers may be documented across many different note types, which creates challenges for cNLP systems to consistently identify all of them. Previous work often focused on specific note types such as radiology reports to simplify system development, with the assumption that certain clinical information often appears in a narrow set of notes.

Second, the characteristics of certain clinical entities make it challenging to extract using cNLP. For example, environmental exposure information was rarely documented in clinical notes in our study. Sparse documentation thus makes it difficult to identify notes that include potentially relevant information for medical experts to annotate. In addition, the documentation of environmental exposure information is highly inconsistent, with many mentions directly naming substances (eg, Agent Orange) as well as alluding to potential exposures through locations (eg, shipyards and combat sites) or deployment (eg, Vietnam or Camp Lejeune). As environmental exposure documentation has not been a routine component in clinical documentation until very recently because of the PACT Act, there are no standards for consistently documenting such information and further compiling a high-quality dataset for training cNLP models to extract them. To the best of our knowledge, this is the first study that set out to develop and use NLP to extract potential military environmental exposures in accordance with the PACT Act, despite prior studies that directly used structured data to infer such exposures [25]. In our future work, we will assess to what extent the NLP-extracted exposures overlap with those inferred from structured data and whether NLP can identify additional exposed patients that structured data misses.

Third, the heterogeneity of clinical information also raises challenges for cNLP systems to accurately identify all relevant information. For example, while our cNLP pipeline showed decent performance in identifying the primary diagnosis, it was unable to identify the myriad of secondary and differential diagnoses for patients’ comorbidities that span a wide range of diseases such as prostate cancer and bladder cancer. Therefore, we prioritized our efforts on optimizing the primary diagnosis rather than secondary and differential diagnoses, which are also underexplored in existing cNLP efforts. In our future work, we will continue to refine the cNLP pipeline to identify the various types of diagnoses from clinical notes, including optimizing for the capture of secondary and differential diagnoses.

Fourth, while LLMs such as Bidirectional Encoder Representations from Transformers and Llama have demonstrated superior performance in many cNLP tasks, they appeared to have poor transferability in our study. This may be due in part to the characteristics of clinical information that were unique to rare cancers such as LMs and how notes were composed within the VA system. A more comprehensive and in-depth evaluation of general and clinical LLMs on clinical notes for rare cancers is therefore needed to assess their performance and transferability and identify ways to adapt them to rare cancers.

### Assessing Biases in Applying NLP on Clinical Notes for Rare Diseases

The results of this study suggest potential biases in cNLP performance in extracting substance use and the primary diagnosis from the notes of non-Hispanic White veterans versus non-Hispanic Black veterans. Several strategies could be used to mitigate such potential biases. First, it may be desirable to use standardized documentation, that is, the type of substance and frequency or duration of exposure, such as “tobacco use, 3 packs per day for 50 years.” Second, researchers may consider alternative sampling methods when selecting notes and patient samples for developing cNLP methods, especially when the patient population is already heavily dominated by patients with certain characteristics, such as older White adults in our patient cohort.

### Clinical Impacts

The PACT Act aims to provide benefits for veterans who have been exposed to environmental toxicants that are associated with cancer diagnosis and progression, but we found that environmental exposure information is only scarcely and inconsistently documented in our study data. We observed that especially for non-Hispanic Black patients, the documentation rate is low. In addition, the ways environmental exposure information was documented also differ, with some being explicitly documented (eg, “exposure to Agent Orange”) and many only alluding to exposures by documenting deployment sites and occupations that may expose veterans to toxicants. Toxic exposure screening was implemented as part of the PACT Act across VA systems nationwide [26], which could help capture environmental exposure information. Our study indicates that more consistent and comprehensive adherence to documentation of environmental exposure is needed in order to facilitate the efficient identification of eligible patients for clinical research, patient care, and benefit enrollment.

There were statistically significant differential performances in the FNs of extracting substance use information from notes for non-Hispanic White versus non-Hispanic Black patients. This may be due to the rich information and variations in documenting patients’ substance use. The use of templates will help standardize how documentation occurs as well as how information is captured. This will also help improve the performance of cNLP extraction pipelines because fewer outliers will appear in the clinical documentation. Such templates will also help standardize the documentation of substance use. Templates that are autopopulated can also capture data more uniformly.

### Limitations

As a preliminary effort for developing a cNLP system for LMs, our study had several limitations. By using nationwide VA EHR data, there was variability in data quality and completeness. As mentioned previously, documentation of elements such as environmental exposures did not have an established standard, resulting in inconsistent and heterogeneous documentation. Military exposure documentation was infrequent, which reflects the nuanced nature of this type of screening. However, the recently mandated screening implemented in August 2023 will improve documentation for future research and analysis. Our study only examined racial bias due to the limited number of annotated notes we had and may have overlooked other potential sources of bias such as age, gender, and geography. Finally, our data come from the national VA health care system. Despite being a single EHR system, the VA health care system spans the entire country as well as Puerto Rico, Guam, and the Philippines, and its EHR captures the heterogeneity in regional practice patterns. For our work in understanding military exposure and the association with developing LMs, it is a vertically integrated system that is unique in the patient population and prioritization of military service–connected conditions, suitable for our research objective.

### Future Directions

Our future directions include improving the performance of the cNLP pipeline. To achieve this, we plan to leverage more advanced pretrained LLMs such as CancerBERT [27] and GatorTron [28] and fine-tune them on our dataset to extract the clinical entities. We will use the rule-based cNLP pipeline developed in this study to generate weak labels for fine-tuning these LLMs, without having medical experts to further annotate notes. We will also conduct a more thorough and large-scale review of the environmental exposure documentation to construct a more comprehensive terminology dictionary for identifying environmental exposures for patients with LMs. In addition, many clinical entities should be extracted in fine-grained forms (eg, substance use with frequency, amount, and status) and in relation to other information if available (eg, temporal information). Another next step is to use cNLP to identify and analyze the current documentation practices and potential biases for less common clinical entities. We will also apply cNLP-enabled analyses to all veterans with LMs in the VA CDW to assess the documentation patterns of the clinical information among different patient groups (by race, gender, branch, and socioeconomic status); provider types; and note types to identify potential biases. We will also apply the pipeline to clinical notes from health systems outside of the VA to assess its generalizability.

Another future direction is to design and implement a tool integrated into the EHR system and help clinicians improve their documentation quality and identify inconsistencies or inadequacies (eg, lack of documentation of environmental exposures) at the time of documentation.

### Conclusions

In our rule-based cNLP pipeline that integrates clinical domain expertise, clinical entities such as performance status, staging, and the primary diagnosis were captured with satisfying accuracy. The primary diagnosis and substance use exhibit differential performance for non-Hispanic Black and White patients. Our future work will build upon the rule-based pipeline and leverage more advanced models to enhance the performance of extraction for substance abuse and environmental exposures, as well as ensuring that model performance is equitable across different racial groups.

## Supplementary material

10.2196/63908 Multimedia Appendix 1 Histology used to retrieve the patient cohort based on *International Classification of Disease for Oncology, Third Edition (ICD-O-3)*.

10.2196/63908 Multimedia Appendix 2 Annotation guideline.

10.2196/63908 Multimedia Appendix 3 Confusion matrices and chi-square test results for clinical entities.

## Abbreviations

CDW: Corporate Data Warehouse
cNLP: clinical natural language processing
ECOG: Eastern Cooperative Oncology Group
EHR: electronic health record
FN: false negative
FP: false positive
HIPAA: Health Insurance Portability and Accountability Act
KPS: Karnofsky performance status
LLM: large language model
LM: lymphoid malignancy
NLP: natural language processing
PACT: Promise to Address Comprehensive Toxics
VA: Veterans Affairs
