# Supplementary material for: Clinical Information Extraction From Notes of Veterans With Lymphoid Malignancies: Natural Language Processing Study
Source: JMIR Med Inform. 2025 Oct 16;13:e63908. doi: 10.2196/63908 (PMC12530692; doi:10.2196/63908)
Supplement: Multimedia Appendix 1 [file medinform-v13-e63908-s001.docx]

Multimedia Appendix 1: Histology used to retrieve the patient cohort based on International Classifications of Disease for Oncology, Third Edition (ICD-O-3).

| 'COMPOSITE HODGKIN AND NON-HODGKIN LYMPHOMA',  'HODGKIN LYMPHOMA, LYMPHOCYTE DEPLETION, NOS',  'HODGKIN SARCOMA',  'HODGKIN LYMPHOMA, LYMPHOCYTE-RICH',  'HODGKIN LYMPHOMA, NOS',  'HODGKIN GRANULOMA',  'HODGKIN LYMPHOMA, LYMPHOCYTE DEPL, RETICULAR',  'HODGKIN LYMPHOMA, NODULAR SCLEROSIS, GRADE 1',  'HODGKIN LYMPHOMA, MIXED CELLULARITY, NOS',  'HODGKIN LYMPHOMA, NODULAR SCLEROSIS, GRADE 2',  'HODGKIN LYMPHOMA, NODULAR SCLEROSIS, NOS',  'HODGKIN LYMPHOMA, NODULAR LYMPHOCYTE PREDOMIN',  'HODGKIN LYMPHOMA, LYMPHOCYTE DEPL, DIFF FIBRO',  'HODGKIN LYMPHOMA, NODUL SCLEROSIS, CELL PHASE',  'MALIGNANT LYMPHOMA, LRGE B-CELL, DIFFUSE, NOS',  'MALIG LYMPHOMA, LRG B-CELL, DIFF, IMMUNO, NOS',  'ALK POSITIVE LARGE B-CELL LYMPHOMA',  'MEDIASTINAL LARGE B-CELL LYMPHOMA',  'PRIMARY EFFUSION LYMPHOMA',  'INTRAVASCULAR LARGE B-CELL LYMPHOMA',  'T-CELL/HISTIOCYTE RICH LARGE B-CELL LYMPHOMA',  'LARGE B-CELL LYMPHOMA ARISING IN HHV8-ASSOCIATED MULTICENTRIC CASTLEMAN DISEASE',  'B-CELL CHRON LYMPHOCYTIC LEUK/SMALL LYMPHOMA', 'PROLYMPHOCYTIC LEUKEMIA, B-CELL TYPE',  'PROLYMPHOCYTIC LEUKEMIA, T-CELL TYPE',  'PROLYMPHOCYTIC LEUKEMIA, NOS',  'MALIGNANT LYMPHOMA, SMALL B LYMPHOCYTIC, NOS',  'PRIMARY CUTANEOUS FOLLICLE CENTRE LYMPHOMA',  'FOLLICULAR LYMPHOMA, GRADE 1',  'FOLLICULAR LYMPHOMA, GRADE 2',  'FOLLICULAR LYMPHOMA, GRADE 3',  'FOLLICULAR LYMPHOMA, NOS',  'MALIGNANT LYMPHOMA, LYMPHOPLASMACYTIC',  'WALDENSTROM MACROGLOBULINEMIA',  'SPLENIC MARGINAL ZONE B-CELL LYMPHOMA',  'MANTLE CELL LYMPHOMA',  'MARGINAL ZONE B-CELL LYMPHOMA, NOS',  'HAIRY CELL LEUKEMIA',  'BURKITT CELL LEUKEMIA',  'BURKITT LYMPHOMA, NOS',  'PLASMABLASTIC LYMPHOMA',  'PLASMACYTOMA, NOS',  'MULTIPLE MYELOMA',  'PLASMA CELL LEUKEMIA',  'IMMUNOGLOBULIN DEPOSITION DISEASE',  'PLASMACYTOMA, EXTRAMEDULLARY',  'T-GAMMA LYMPHOPROLIFERATIVE DISEASE',  'MYCOSIS FUNGOIDES',  'MATURE T-CELL LYMPHOMA, NOS',  'SUBCUTANEOUS PANNICULISTIC T-CELL LYMPHOMA',  'HEPATOSPLENIC GAMMA-DELTA CELL LYMPHOMA',  'ADULT T-CELL LEUKEMIA/LYMPHOMA (HTLV-1 POS)',  'PRIM CUTANEOUS CD30+ T-CELL LYMPHOPROLIF DIS (PRE-2021 CASES)', 'ANAPLASTIC LRG CELL LYMPH, T & NULL CELL TYPE',  'INTESTINAL T-CELL LYMPHOMA',  'SEZARY SYNDROME',  'CUTANEOUS T-CELL LYMPHOMA, NOS',  'NK/T-CELL LYMPHOMA, NASAL AND NASAL-TYPE',  'PRIM CUTANEOUS CD30+ T-CELL LYMPHOPROLIF DIS',  'T-CELL LARGE GRANULAR LYMPHOCYTIC LEUKEMIA',  'AGGRESSIVE NK-CELL LEUKEMIA',  'CHRONIC LYMPHOPROLIFERATIVE DISORDER OF NK-CELLS',  'PRIMARY CUTANEOUS GAMMA-DELTA T-CELL LYMPHOMA',  'T-CELL LARGE GRANULAR LYMPHOCYTIC LEUKEMIA',  'LYMPHOID LEUKEMIA, NOS',  'LYMPHOPROLIFERATIVE DISORDER, NOS',  'MALIGNANT LYMPHOMA, NOS',  'MALIGNANT LYMPHOMA, NON-HODGKIN, NOS' |
| --- |
