# Supplementary material for: Clinical Information Extraction From Notes of Veterans With Lymphoid Malignancies: Natural Language Processing Study
Source: JMIR Med Inform. 2025 Oct 16;13:e63908. doi: 10.2196/63908 (PMC12530692; doi:10.2196/63908)
Supplement: Multimedia Appendix 2 [file medinform-v13-e63908-s002.docx]

**Multimedia Appendix 2: Performance of extracting Staging using different window sizes**

Window size = 70

|  | Precision | Recall | F1 |
| --- | --- | --- | --- |
| Test set 1 | 0.82 | 0.79 | 0.81 |
| Test set 2 | 0.71 | 0.66 | 0.68 |

Window size = 50

|  | Precision | Recall | F1 |
| --- | --- | --- | --- |
| Test set 1 | 0.85 | 0.73 | 0.79 |
| Test set 2 | 0.78 | 0.64 | 0.70 |

**Window size = 30 (reported in the study)**

|  | Precision | Recall | F1 |
| --- | --- | --- | --- |
| Test set 1 | 0.94 | 0.69 | 0.80 |
| Test set 2 | 0.85 | 0.60 | 0.71 |

Window size = 20

|  | Precision | Recall | F1 |
| --- | --- | --- | --- |
| Test set 1 | 0.95 | 0.62 | 0.75 |
| Test set 2 | 0.88 | 0.55 | 0.68 |

Window size = 10

|  | Precision | Recall | F1 |
| --- | --- | --- | --- |
| Test set 1 | 0.94 | 0.48 | 0.64 |
| Test set 2 | 0.87 | 0.35 | 0.50 |
