# Supplementary material for: Clinical Information Extraction From Notes of Veterans With Lymphoid Malignancies: Natural Language Processing Study
Source: JMIR Med Inform. 2025 Oct 16;13:e63908. doi: 10.2196/63908 (PMC12530692; doi:10.2196/63908)
Supplement: Multimedia Appendix 3 [file medinform-v13-e63908-s003.docx]

**Multimedia Appendix 3: Confusion Matrices and Chi-Square Test Results for Clinical Entities**

Performance Status

# FP: X-squared = 3.1119, df = 1, p-value = 0.07772, 95% CI (0.0024, 0.216)

# FN: X-squared = 0.013521, df = 1, p-value = 0.9074

# FN (Fisher’s Exact Test): 95% CI (0.1229, 13.771), p-value = 1

|  | Black | White |
| --- | --- | --- |
| FP | 12 | 2 |
| FN | 3 | 2 |
| TP | 70 | 52 |

Staging (Window size = 30)

# FP: X-squared = 1.7028, df = 1, p-value = 0.1919, 95% CI (-0.026, 0.204)

# FN: X-squared = 0.61787, df = 1, p-value = 0.4318, 95% CI (-0.082, 0.216)

|  | Black | White |
| --- | --- | --- |
| FP | 12 | 3 |
| FN | 46 | 22 |
| TP | 70 | 49 |

Primary diagnosis

**# FP: X-squared = 10.219, df = 1, p-value = 0.00139, 95% CI (0.04, 0.163)**

**# FN: X-squared = 10.388, df = 1, p-value = 0.001268, 95% (0.036, 0.128)**

|  | Black | White |
| --- | --- | --- |
| FP | 273 | 109 |
| FN | 73 | 19 |
| TP | 412 | 258 |

Substance use

# FP: X-squared = 0.00042264, df = 1, p-value = 0.9836, 95% CI (-0.136, 0.117)

**# FN: X-squared = 5.8699, df = 1, p-value = 0.0154, 95% CI (-0.276, -0.026)**

|  | Black | White |
| --- | --- | --- |
| FP | 58 | 34 |
| FN | 36 | 41 |
| TP | 112 | 63 |

Environmental exposure

# FP (Fisher’s Exact Test): 95% CI (0.267, 23.5), p-value = 0.3578

# FN (Fisher’s Exact Test): 95% CI (0.079, 11.156), p-value = 1

|  | Black | White |
| --- | --- | --- |
| FP | 3 | 7 |
| FN | 2 | 11 |
| TP | 3 | 18 |
